# p53/PCDH17/Beclin-1 Proteins as Prognostic Predictors for Urinary Bladder Cancer

**DOI:** 10.7150/jca.37335

**Published:** 2019-10-15

**Authors:** Liuxi Chen, Ying Liu, Qi Zhang, Mingming Zhang, Xuemeng Han, Qiujie Li, Tian Xie, Qibiao Wu, Xinbing Sui

**Affiliations:** 1 Department of Medical Oncology, Sir Run Run Shaw Hospital, College of Medicine, Zhejiang University, Hangzhou, Zhejiang, China; 2 Holistic Integrative Pharmacy Institutes and Comprehensive Cancer Diagnosis and Treatment Center, the Affiliated Hospital of Hangzhou Normal University, College of Medicine, Hangzhou Normal University, Hangzhou, Zhejiang, China; 3 Key Laboratory of Elemene Class Anti-cancer Chinese Medicine of Zhejiang Province and Engineering Laboratory of Development and Application of Traditional Chinese Medicine from Zhejiang Province, Hangzhou Normal University, Hangzhou, Zhejiang, China; 4 Department of Urology and Institute of Department of Urology, Zhejiang Provincial People's Hospital, Hangzhou Medical College, Hangzhou, Zhejiang, China; 5 State Key Laboratory of Quality Research in Chinese Medicines, Faculty of Chinese Medicine, Macau University of Science and Technology, Macau, P.R. China

**Keywords:** p53, protocadherin 17, Beclin-1, Urinary bladder neoplasms

## Abstract

**Purpose:** To determine whether p53, PCDH17, Beclin-1 expression is associated with clinicopathological characteristics of bladder cancer.

**Materials and Methods:** 75 patients with non-muscle-invasive and muscle-invasive bladder cancer were included. Immunohistochemical staining for p53, PCDH17 and Beclin-1 were carried out on the same paraffin-embedded blocks serial sections of these patients who underwent surgery between 2010 and 2015. In addition, p53 gene mutations in these tumors were screened by DNA sequencing.

**Results:** Forty-nine (66.7%) of 75 tumors had p53 gene mutations detected by DNA sequencing method. Of these tumors, 43 (86.0%) exhibited p53 high expression. Furthermore, p53 mutation and low expression of PCDH17 were significantly associated with muscle-invasive bladder cancer. Beclin-1 was also strongly associated with T stage. The p53 mutation, the expression of p53 and PCDH17 were significantly associated with survival from bladder cancer. In addition, patients with p53 high-expression or p53 mutation, PCDH17 low-expression and Beclin-1 low-expression significantly had a poor prognosis.

**Conclusions:** Use of a DNA sequencing method to detect p53 gene mutations was consistent with an immunohistochemical method to detect p53 alterations. In conjunction with levels of p53/PCDH17/Beclin-1, p53 and PCDH17 were independently associated with prognosis; Beclin-1 only had a tendency towards overall survival. p53/PCDH17/Beclin-1 phenotype seems to play a more important role than p53 expression in bladder cancer outcome. It is also identified that p53/PCDH17, p53/Beclin-1 or PCDH17/Beclin-1 all have a cooperative and synergistic effect, which may provide us the potential biomarker for bladder cancer patients.

## Introduction

Urinary bladder cancer (UBC) is the fourth most frequent malignancy in men in the United States 1. To the best of our knowledge, 90% of urinary bladder cancers are urothelial cell carcinomas, which are broadly categorized into muscle-invasive bladder cancer (MIBC) and non-muscle-invasive bladder cancer (NMIBC) 2. MIBC presents with a poor prognosis, and this definition represents more than 50% mortality account for their disease 3. Patients with NMIBC generally have a variable clinical course with potential for progression and significant risk of recurrence 4. The International Society of Urological Pathology (ISUP) meeting in 2013 declared that there was no ideal marker in the respect of urothelial differentiation 5. In recent years, there have been great effort in biomarkers in the prognosis and prediction of bladder cancer (BLCA), such as protein 53 (p53), protein 21 (p21), RB transcriptional corepressor (pRB), survivin and so on 6. Even more interesting is the probability of finding precise biomarkers that could be applied to routine clinical practice to evaluate clinical outcome by immunohistochemistry 7.

Urinary bladder cancer, a heterogeneous disease, develops via two pathways referred to as basal and luminal subtype which correspond to different clinical behaviors and distinct responses to chemotherapy 8. And the emerging evidence indicates the complex structural genomic alterations with the dysregulation of several key regulatory pathways involving cell cycle, phosphatidylinositol 3 kinase (PI3K) signaling, and chromatin remodeling 9. The most frequently mutated gene is tumor protein p53 (TP53) in MIBC, which acts upon cell-cycle progression. A growing number of studies suggest that both p53 and phosphatase and tensin homologue deleted on chromosome 10 (PTEN) deficiency contribute to the development of an invasive phenotype of UBC 10. p53 expression is more frequently strong in higher stage of urothelial carcinoma 11, 12. Interestingly, autophagy is strictly interconnected with the bladder cancer progression 13. Beclin-1, an important mediator of autophagy, correlated with Bcl-2 plays a critical role in the development and progression of UBC 14. Fortunately, fundamental insights into the biology of UBC are starting to emerge, based on genetic and epigenetic changes 15. DNA methylation, histone modification and RNA interference are well-established epigenetic alterations in bladder cancer 16. What's more, DNA methylation leads to the inactivation of tumor suppressor genes and to the potential for diagnosis, prognosis and therapy of bladder cancer 17. Protocadherin 17 (PCDH17), belonging to the subgroup of the cadherin superfamily, is frequently silenced by promoter methylation 18. PCDH17 promoter methylation is closely related with malignant behavior and may be regarded as an independent predictor of clinical outcomes in bladder cancer 19. Costa et al. found that the correlation between PCDH17 methylation and clinicopathological parameters (such as pathologic stage and grade) provide the descriptions of prognosis in bladder cancer 18.

Taken together, it has been acknowledged that mutant p53 may gain functions that accelerate malignant progression and increase cancer invasiveness through disordered autophagy regulation. The recent findings that p53 overexpression contributes to the inhibition of Beclin-1 ubiquitination, which is associated with high autophagic activity 20. Disordered autophagy regulation and imbalanced cell growth via p53/Bcl-2/Beclin-1 pathway lead to accumulation of cells in glioma 21. Studies investigating p53/Bcl-2/Beclin-1 pathway with clinicopathological parameters in urinary bladder cancer have been conflicting. What's more, the interaction of p53 and PCDH17 in bladder cancer prognosis remains unknown.

Here, we retrospectively selected 75 urinary bladder cancer cases and performed immunohistochemical analysis for p53, PCDH17 and Beclin-1 on the same paraffin-embedded blocks. We also compared p53 gene mutations using immunohistochemical analysis with that of DNA-based sequence analysis. The purpose of the present study is to determine that p53/PCDH17/Beclin-1 is as part of an effort to predict urinary bladder cancer progression and prognosis.

## Materials and Methods

### Patient population and tumor samples

All patient information and clinical data were obtained from Sir Run Run Shaw Hospital, Zhejiang University, Hangzhou, China. A total of 75 patients with formalin-fixed, paraffin-embedded tumors (FFPE) were selected retrospectively, who had undergone surgery between 2010 and 2015. Patients were histopathologically diagnosed by pathology department. We excluded patients who had received preoperative radiotherapy, chemotherapy, or chemoradiotherapy. Histology, grade, stage, and presence of carcinoma in situ were confirmed by pathology department in Sir Run Run Shaw Hospital. Tumor staging and grading was carried out according to the American Joint Committee on Cancer (AJCC), TNM Staging System for Urethral Carcinoma (8th ed., 2016). When the patient died, the cause of death was determined by the attending physician or by death certificate.

### Immunohistochemistry

p53, PCDH17 and Beclin-1 immunohistochemistry (IHC) were available in 75 cases. Each case was selected from a hematoxylin-eosin (H&E)-stained section of a donor block and performed p53, PCDH17 and Beclin-1 immunohistochemical staining using the aboving sections from the same paraffin-embedded blocks. The sections were incubated with p53 antibody (1:750 dilution; DO-1, SANTA CRUZ, Europe), PCDH17 antibody (1:1000 dilution; HPA026817, Sigma, CA, USA) and Beclin-1 (1:150 dilution; E-8, SANTA CRUZ, Europe) respectively overnight at 4 °C. A DAB Kit (GK300710, GENE TECH, CHINA) was applied to the sections, followed by haematoxylin counterstain.

Comparing cancer tissue with paracancer tissue, the staining conditions for each antibody were established, which was used as internal control in each staining protocol. Specimens from normal tissue (for PCDH17 and Beclin-1) and bladder cancer tissue (for p53) served as positive controls. In p53 IHC, nuclear expression with higher intensity than internal positive control was regarded as positive. Cytoplasmic staining in 75 cases with higher intensity than internal positive control was interpreted as positive in PCDH17 or Beclin-1 IHC. In each case, at least 500 cells were evaluated in 5 representative high power fields (200 ×), and the proportion of positively stained cells was calculated. According to the p53, PCDH17 and Beclin-1 protein staining, protein expression was scored based on the intensity of nuclear or cytoplasmic staining, using a four-point system: 0, negative; 1, weak; 2, moderate; and 3, strong. All immunostaining score were achieved by two investigators who were blinded to clinical data. Then, we divided patients into two groups: low expression (0 and 1, -) or high expression (2 and 3, +).

### p53 mutation analyses

In the Molecular Pathology Laboratory of Department of Pathology, Sir Run Run Shaw Hospital, Zhejiang University, Hangzhou, China, assessment of p53 mutational status was using appropriate quality control procedures. Mutation status was determined using genomic DNA extracted from tumor tissue. P53 mutation analysis was carried out using bi-directional Sanger sequencing analysis performed on an independent polymerase chain reaction (PCR) and primers. The sequencing was done on 3730XL with BigDye Taq FS Terminator version 3.1 with analysis done on an ABI Sequence Scanner version 1.0.

### Statistical analyses

For purposes of analysis, tumor stage (Ta, Tis, T1 vs. T2, T3, T4), stage (stage0a, 0is, I vs. stage II, III, IV) were evaluated as dichotomized variables. The two-tailed χ2 test was used to analyse the association of protein expression with clinicopathological parameters. The relationship with patient survival was analyzed through Kaplan-Meier analysis, which was assessed with the log-rank test. Multivariate survival analysis was performed by Cox proportional hazards regression model. For all tests, p < 0.05 was considered statistically significant. All analyses were performed with SPSS 11.0 for Windows (SPSS Inc., Chicago, IL, USA).

## Results

### Expression of p53, PCDH17 and Beclin-1 in urinary bladder cancer

**Figure 1:**
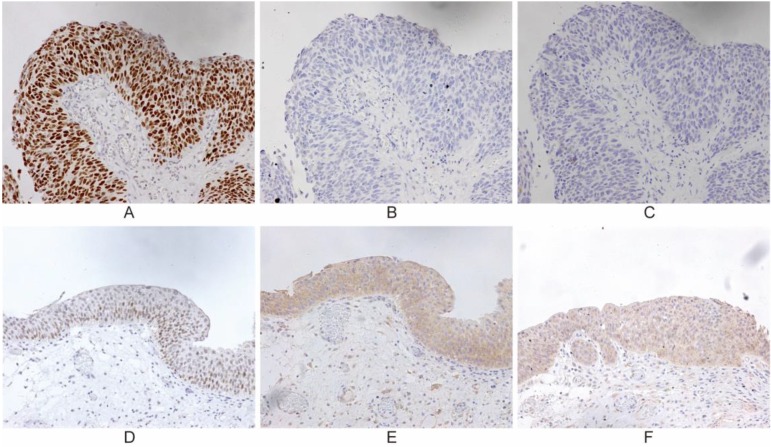
Representative immunohistochemical staining for p53, PCDH17 and Beclin-1 in urinary bladder cancer, which is expressed in the nuclear or cytoplasm (200 ×). Case 1: p53 mutation/ p53 wild-type **(A, D)**; Case 2: PCDH17 mutation/ PCDH17 wild-type **(B, E)**; Case 3: Beclin-1 mutation/ Beclin-1 wild-type **(C, F)**.

IHC was carried out in 75 specimens to examine the expression of p53, Beclin-1 and PCDH17 in UBC patients who underwent surgical treatment (cystectomy, transurethral resection of bladder tumor), which was selected regarding to the AJCC guideline (Table 1). Normal bladder epithelium showed heterogeneous Beclin-1, heterogeneous PCDH17 and absent p53. Beclin-1 and PCDH17 staining patterns were mainly cytoplasmic, while p53 was in the nuclear (Figure 1). We also detected alterations in the p53 gene by means of the DNA-based sequencing method. Fifty (66.7%) of 75 tumors in urinary bladder cancer had p53 gene mutations. Forty-three tumors (86.0% of the p53 mutant) had elevated levels of p53 protein as detected by IHC (P<0.001, Table 1), indicating a 12.2% false-positive frequency with IHC. Thus, tumors from 75 patients were available for this comparative study.

Next, we examined the correlation of these three markers. Forty-eight tumors showed absolutely inverse staining between p53 and PCDH17, while 51 between p53 and Belin-1. PCDH17 staining was strongly concordant with Beclin-1 expression in all tumors (54/75, concordance rate, 72.0%). For further analyses, we combined the three markers. Twenty-nine tumors that were p53 high expression and inversely showed low expression of Becin-1 staining (Pearson χ2, p < 0.05). There was a similar relationship between PCDH17 and Beclin-1, with thirty tumors showing weak PCDH17 staining and also low expression of Beclin-1 staining (Pearson χ2, p < 0.05).

The p53 mutation and the expression of PCDH17 were strongly associated with MIBC (Pearson χ2, p < 0.001, respectively). There was also statistical significance in association with tumor stage and pathological stage (Pearson χ2, p < 0.05).

### Association of p53, PCDH17, and Beclin-1 as individual variable with patient survival

**Figure 2:**
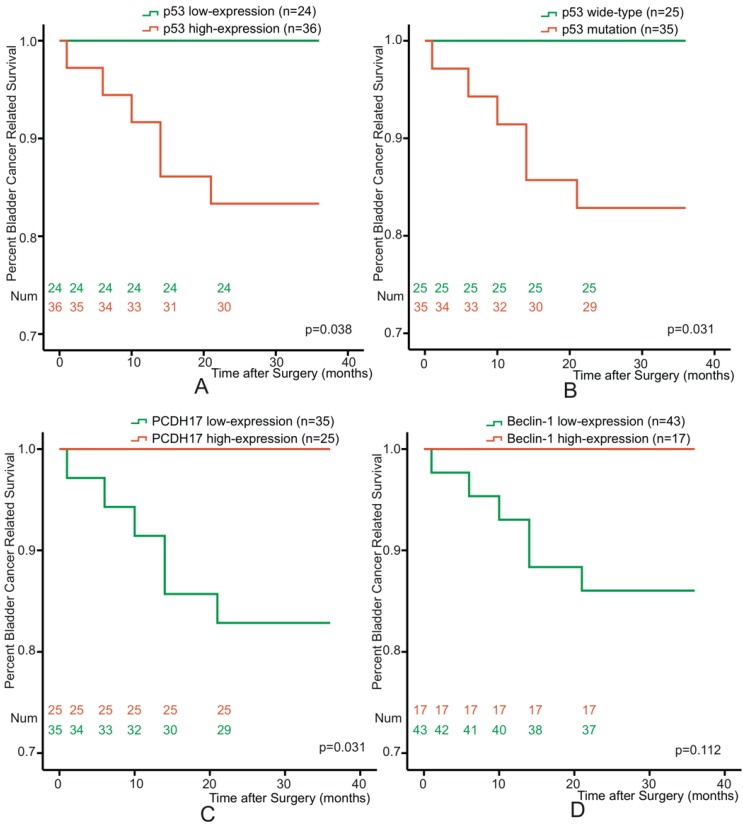
Kaplan-Meier survival curves of 60 Bladder Cancer patients who underwent surgical treatment according to p53 mutation and p53, PCDH17, Beclin-1 expression. **(A)** The overall survival rate for patients with p53 low-expression was significantly higher than that for patients with p53 high-expression (p=0.038). **(B)** The overall survival rate for patients with p53 mutation was significantly higher than that for patients with p53 wide-type (p=0.031). **(C)** The overall survival rate for patients with PCDH17 high-expression was significantly higher than that for patients with PCDH17 low-expression (p=0.031). **(D)** No significant difference between the overall survival rate for patients with Beclin-1 high-expression and that for patients with Beclin-1 low-expression (p=0.112).

To determine the association between p53, PCDH17, or Beclin-1 and prognosis of bladder cancer patients, all patients were followed-up disease outcomes after surgery. The followed-up period was 36 months or more, and 11 patients were dead at the time of analysis. Re-operation occurred in 3 patients, and two of them were dead after radical cystectomy. We excluded the characteristic of tumor stage, and selected 60 non-muscle invasive bladder cancer patients. Kaplan-Meier analyses were performed with respect to patient survival from bladder cancer for p53 mutation or p53, PCDH17 and Beclin-1 expression. p53 and PCDH17 were each significantly associated with survival from bladder cancer (p=0.038, p=0.031, p=0.031, Figure 2A, 2B, 2C), while low-expression of Beclin-1 just showed a trend towards patient survival (p=0.112, Figure 2D).

### Association of p53, PCDH17, and Beclin-1 as combined variable with patient survival

**Figure 3:**
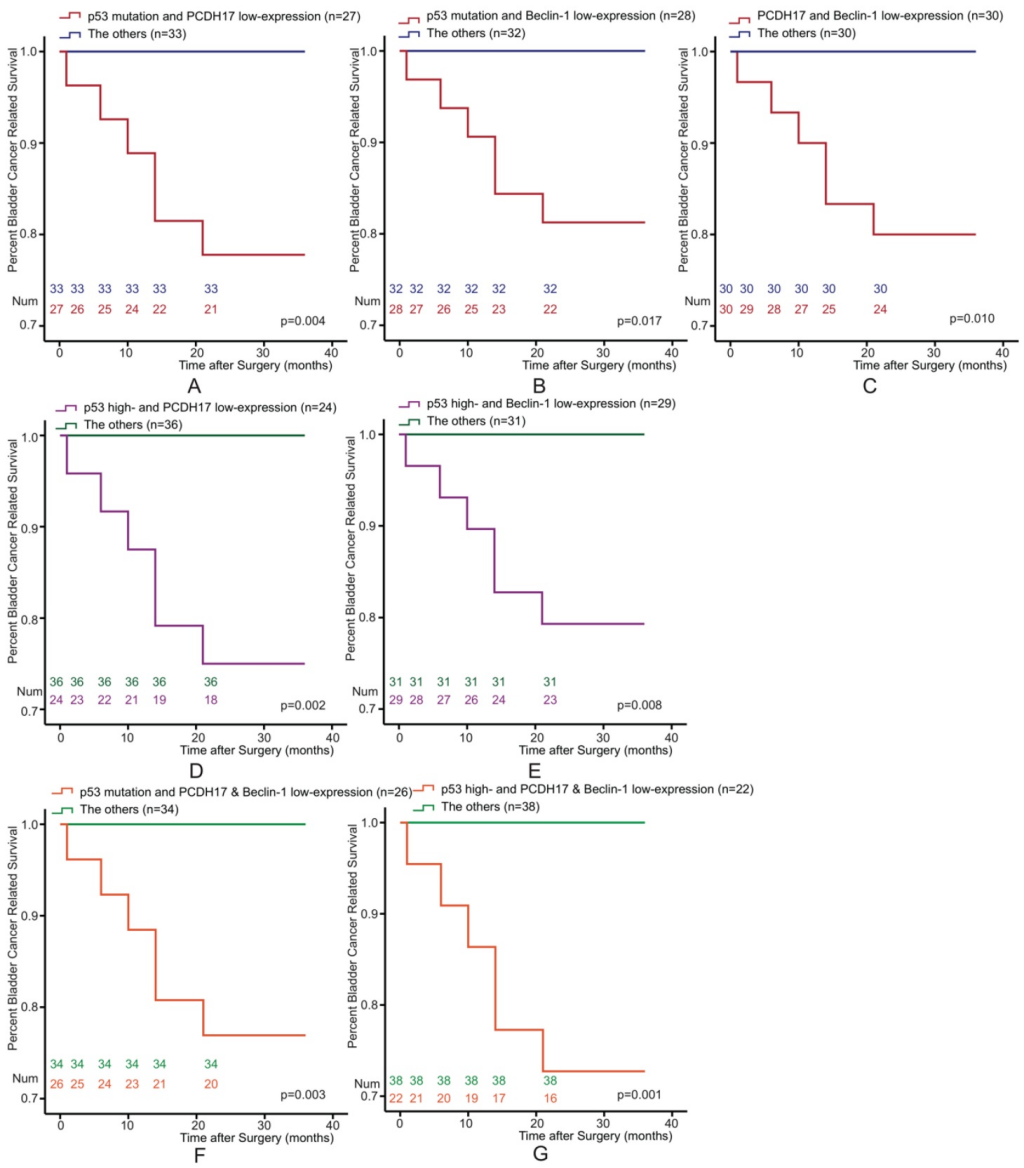
Kaplan-Meier survival curves of 60 Bladder Cancer patients who underwent surgical treatment according to combined p53, PCDH17 and Beclin-1 expression, which was classified it into two types. **(A)** The overall survival rate for patients with p53 mutation plus PCDH17 expression (p=0.004). **(B)** The overall survival rate for patients with p53 mutation plus Beclin-1 expression (p=0.017). **(C)** The overall survival rate for patients with PCDH17 expression plus Beclin-1 expression (p=0.010). **(D)** The overall survival rate for patients with combined p53 and PCDH17 expression (p=0.002). **(E)** The overall survival rate for patients with combined p53 and Beclin-1 expression (p=0.008).** (F)** The overall survival rate for patients with combined p53 mutation and PCDH17 plus Beclin-1 expression (p=0.003). **(G)** The overall survival rate for patients with combined p53 expression and PCDH17 plus Beclin-1 expression (p=0.001).

The Kaplan-Meier survival curve of BLCA patients also be estimated according to the following three potential combinations of combined variables: p53 and PCDH17, p53 and Beclin-1, and PCDH17 and Beclin-1. We classified it into four types (low-/high-expression, both high-expression, high-/low-expression, both low-expression). Patients could be stratified as low- and high-risk for death. We found that the combined p53 and PCDH17 status was associated with bladder cancer survival. Combination p53 mutation or p53 high-expression with PCDH17 low-expression versus the others were significantly association with survival for BLCA (p=0.004, p=0.002, respectively, Figure 3A, 3D). Similarly, p53/Beclin-1 (p=0.017, p=0.008, Figure 3B, 3E) and PCDH17/Beclin-1 staining status (p=0.010, Figure 3C) were also associated with bladder cancer survival.

Of the 60 patients, 22 of them were p53 high-expression and PCDH17 plus Beclin-1 low-expression status, while twenty-six of them with p53 mutation and PCDH17 plus Beclin-1 low-expression status. When analyzed as categoric variables in Kaplan-Meier analysis, the bladder cancer had a poor prognosis (p=0.003, p=0.001, Figure 3F, 3G).

### Cox analysis of combined variables incorporating pathologic features

Univariate analysis showed that stages (p=0.031), p53 mutation (p=0.157), p53 expression (p=0.148) and PCDH17 expression (p=0.140) were associated with overall survival rates (Table 2). We also confirmed that the combined p53 and Beclin-1 status was associated with bladder cancer survival (p=0.038). So was PCDH17/Beclin-1 (p=0.038). When overall p53, PCDH17 and Beclin-1 status as a single marker, it was significantly associated with bladder cancer survival (p=0.008).

According to the data above, we comprised p53, PCDH17 expression and Beclin-1 expression. For multivariable Cox proportional hazards regression analysis, stages, p53/PCDH17/Beclin-1 expression were adjusted for the whole group of tumors (Table 3). When p53/PCDH17/Beclin-1 expression was modeled with stages, it was the sole predictors of bladder cancer death (p=0.015). We have a preliminary conclusion that: p53 high-expression plus PCDH17 low-expression and Beclin-1 low-expression were significantly in connection with a poor prognosis.

## Discussion

The tumor suppressor gene TP53 serves as a 'genome guardian', maintaining genomic integrity and stability by triggering cell-cycle arrest, DNA repair, and apoptosis, as well as autophagy 22. Complex molecular circuitries contribute to Bladder tumorigenesis, relying on TP53 inactivation and and tumor-suppressor dysfunction 6. p53 has been studied as a marker to determine the risk of urothelial cell carcinoma recurrence and progression 23. Wild-type p53 prevents its accumulation in the cell nucleus due to the short half-life of up to 30 min for its detection by immunohistochemistry 24. However, TP53 mutations result in increased intra-nuclear p53 accumulation which are resistant to ubiquitin-mediated degradation. Despite the large body of evidence indicating a progressive increase of p53 IHC expression from non-missense mutations (i.e. nonsense, deletion, and insertion), to wild-type TP53, and eventually to inactivating mutations 25. Significant correlations are observed between the invasiveness of urothelial cell carcinoma and the immunohistochemical patterns of TP53 mutations, but single p53 marker is not sufficient to assess outcome of MIBC. It is exactly that not fully understood 6. There is no consensus on which antibody is most suitable for the assessment of mutation-associated p53 expression 26. Nenutil R et al. verified that the phospho-specificity of the antibody did not add sufficient value to the outcome prediction comparing with total p53 levels 27. In keeping with previous papers, we demonstrate that there is also p53 high-expression at the protein level in urinary bladder cancer. What's more, p53 overexpression is correlated with high stage and invasiveness, which refer to an aggressive tumor phenotype.

Mutant p53 proteins, not wild type p53, suppresses the autophagic flow from autophagy related gene (ATG) regulation to the autophagic vesicles formation and their fusion with lysosomes 28. In addition, p53 binding to the adenosine monophosphate activated protein kinase α (AMPKα) subunit counteracts AMPK complex with the concomitant phosphorylation of upstream kinases 29. The activation of the phosphatase and tensin homologue deleted on chromosome 10/ phosphatidylinositol 3 kinase/ protein kinase B/ mammalian target of rapamycin (PTEN/PI3K/AKT/mTOR) pathway can also induce the tumorigenic phenotype 10. Stimulation of mTOR pathway has been associated with the repression of Beclin-1 phosphorylation, which activates the autophagy related protein 14L- vacuolar protein sorting (ATG14L-VPS34) (a class III phosphatidylinositol 3 kinase) complex 30. Liu et al. reported Beclin-1 expression decreased in bladder cancer tissues, which was found in the mRNA level, protein expression, and immunoreactivity 14. They also showed that the expression level of Beclin-1 is related with histopathological grades, TNM stages and overall survival 14. As has been suggested by some articles, down-regulation of Beclin-1 may play a key role in aggressiveness and progression by deregulation of autophagy 31. Also, the Beclin-1 expression has been shown to be inversely associated with the expression of Bcl-2 in bladder cancer by regulation of autophagy 31. The recent findings that p53 inhibits Beclin-1 ubiquitination results in the high level of Beclin-1, which shows high autophagic activity 20. Disordered autophagy regulation and imbalanced cell growth via p53/Bcl-2/Beclin-1 pathway lead to accumulation of cells in glioma 21. However, p53-Beclin-1 interaction in UBC remains unknown. Our results show that alterations of Beclin-1 have a tendency for muscle-invasive bladder cancer and tumor stages. However, Beclin-1 only has a trend for bladder cancer survival, not significant association.

Protocadherins (PCDHs), belonging to the cadherin superfamily of proteins, are divided into two groups: clustered and non-clustered 32. In recent years, PCDH17 was identified as a tumor suppressor gene for breast cancer, through promoter methylation 33. However, little is known about the role of PCDH17 in urothelial bladder cancers. PCDH17 promoter methylation was significantly correlated with advanced stage and high grade tumors, revealing that PCDH17 might therefore be a prognostic target in bladder cancer 19. In addition, DNA methylation, which induces the inactivation of tumor suppressor genes, plays an important role in bladder cancer 16. The most significant new developments are focused on forerunner genes, mainly by hypermethylation, which occur in the early phases of carcinogenesis 8. In our study, we confirm that PCDH17 is also a strong predictor of bladder cancer outcome in patients undergoing surgery. These findings suggest that PCDH17 low-expression is also associated with an aggressive tumor phenotype, involving stages and invasiveness.

Our results showed that a DNA sequencing method to detect p53 gene mutations in bladder cancer was consistent with an immunohistochemical method to detect p53 alterations. p53 and PCDH17 were independently associated with urinary bladder cancer outcomes, while Beclin-1 only had a tendency towards overall survival. p53 high-expression plus PCDH17 and Beclin-1 low-expression were the most significantly in connection with a poor prognosis. The association of the p53/ PCDH17 phenotype with bladder cancer outcome was stronger than p53/Beclin-1 and PCDH17/Beclin-1 phenotype. In our study, we identified that altered expression of p53, PCDH17, and Beclin-1 is associated with bladder cancer survival after adjusting for the effects of pathologic stage, aggressive types. This is the first study, to our knowledge, to refer to the different mechanisms, including autophagy and methylation, in UBC patients who underwent surgery and had long-term follow-up. In short, p53 is the strongest predictor of urinary bladder cancer in patients undergoing surgery. p53/PCDH17 phenotype seems to play a more important role than p53 expression in bladder cancer outcome in patients undergoing surgery. It is also identified that p53/PCDH17, p53/Beclin-1 or PCDH17/Beclin-1 all has a cooperative and synergistic effect, which can stratify patients into two risk groups. The role of these three markers seems to be complex alterations of genetics and epigenetics. Moreover, the concrete pathways remain to be elucidated.

## Conclusions

Our study indicated that p53/PCDH17/Beclin-1 proteins were determined as part of an effort to predict urinary bladder cancer progression and prognosis. These findings should be estimated in larger, multicenter, prospective trials and considered as stratification variables involving urinary bladder cancer patients in clinical trials.

## Figures and Tables

**Table 1:** Association of the Expression of p53, PCDH17, and Beclin-1 With Clinicopathologic Characteristics and Survival Estimates of 75 Bladder Cancer Patients Who Underwent Surgical Treatment.

Characteristic	Patients		p53 Mutation				p53 Expression				PCDH17 Expression				Beclin-1 Expression			
	No.	%	Mutation		Pearson Correlation	p-value	High-expression		Pearson Correlation	p-value	Low-expression		Pearson Correlation	p-value	Low-expression		Pearson Correlation	p-value
			No.	%			No.	%			No.	%			No.	%		
Total	75	100	50	66.7			49	65.3			48	64.0			57	76.0		
Gender					0.115	0.319			-0.037	0.746			-0.016	0.888			-0.165	0.154
Female	16	21.3	9	56.3			11	68.8			10	62.5			10	62.5		
Male	59	78.7	41	69.5			38	64.4			38	64.4			47	79.7		
Smoking					-0.019	0.867			0.003	0.979			-0.025	0.828			-0190	0.100
Yes	29	38.7	19	65.5			19	65.5			19	65.5			25	86.2		
No	46	61.3	31	60.9			30	65.2			29	63.0			32	69.6		
Alcohol					-0.108	0.348			-0.027	0.818			-0.054	0.642			-0.040	0.728
Yes	19	25.3	11	57.9			12	63.2			13	68.4			15	78.9		
No	56	74.7	39	69.6			37	66.1			35	62.5			42	75.0		
Diabetes					0.087	0.451			0.097	0.403			-0.021	0.859			-0.111	0.334
Yes	9	12.0	7	77.8			7	77.8			6	66.7			8	88.9		
No	66	88.0	43	65.2			42	63.6			42	63.6			49	74.2		
BMI					-0.097	0.399			-0.017	0.883			0.110	0.340			0.147	0.203
<24	47	62.7	33	70.2			31	66.0			32	68.1			38	80.9		
>=24	28	37.3	17	60.7			18	64.3			16	57.1			19	67.9		
Tumor stage					0.354	0.002^**^			0.224	0.052			-0.236	0.041^*^			-0.203	0.079
Ta, Tis, T1	60	80.0	35	58.3			36	60.0			35	58.3			43	71.7		
T2, T3, T4	15	20.0	15	100			13	86.7			13	86.7			14	93.3		
Invasiveness					0.354	0.002^**^			0.224	0.052			-0.236	0.041^*^			-0.203	0.079
Invasive	15	20.0	15	100			13	86.7			13	58.3			14	93.3		
Non-Invasive	60	80.0	35	58.3			36	60.0			35	86.7			43	71.7		
p53 Mutation									0.614	<0.001^**^			-0.471	<0.001^**^			-0.530	<0.001^**^
Wide type	25	33.3					6	24.0			8	32.0			11	44.0		
Mutation	50	66.7					43	86.0			40	80.0			46	92.0		
p53 expression					0.614	<0.001^**^							-0.212	0.066			-0.247	0.033^*^
Low	26	34.7	7	26.9							13	50.0			16	61.5		
High	49	65.3	43	87.8							35	71.4			41	83.7		
PCDH17 expression					-0.471	<0.001^**^			-0.212	0.066							0.359	0.002^**^
Low	48	64.0	40	83.3			35	72.9							42	87.5		
High	27	36.0	10	37.0			14	51.9							15	55.6		
Beclin-1 expression					-0.530	<0.001^**^			-0.247	0.002^**^			0.359	0.002^**^				
Low	57	76.0	46	80.7			41	71.9			42	64.0						
High	18	24.0	4	22.2			8	44.4			6	33.3						

*p-value<0.05; ** p-value<0.01

**Table 2:** Univariate Cox Regression Analyses of Pathologic Features and Molecular Markers for the Disease-Specific Survival of 75 Bladder Cancer Patients Who Underwent Surgical Treatment.

Variables	Univariate		
	Hazard Ratio	95%CI	p-value
Gender (male/female)	1.410	0.374-5.315	0.612
Smoking (yes/no)	1.344	0.692-2.609	0.383
Alcohol (yes/no)	0.780	0.422-1.442	0.428
Diabetes (yes/no)	1.193	0.427-3.334	0.737
BMI (<24/>=24)	1.004	0.543-1.856	0.989
TNM Stage (Ta+Tis+T1/T2+T3+T4)	0.519	0.287-0.940	0.031^*^
p53 mutation (mutation/wide type)	0.156	0.012-2.040	0.157
p53 expression (low/high)	0.153	0.012-1.946	0.148
PCDH17 expression (low/high)	6.669	0.538-82.714	0.140
Beclin-1 expression (low/high)	1.848	0.661-5.166	0.242
p53 mutation/PCDH17 expression	0.015	0.000-1.946	0.091
p53 mutation/Beclin-1 expression	0.143	0.018-1.115	0.063
PCDH17/Beclin-1 expression	8.848	1.132-69.145	0.038^*^
p53 mutation/PCDH17/Beclin-1 expression	0.022	0.011-0.702	0.090
p53/PCDH17 expression	0.011	0.000-1.536	0.073
p53/Beclin-1 expression	0.107	0.014-0.834	0.033^*^
p53/PCDH17/Beclin-1 expression	0.063	0.008-0.493	0.008^*^

**Table 3:** Multivariable Cox Regression Analyses of Pathologic Features and Molecular Markers for the Disease-Specific Survival of 75 Bladder Cancer Patients Who Underwent Surgical Treatment.

Variables	Multivariate		
	Hazard Ratio	95%CI	p-value
Model 1			
Stage (0a+0is+I/II+III+IV)	0.458	0.138-1.522	0.202
p53/PCDH17/Beclin-1 expression	0.075	0.009-0.602	0.015^*^
Model 2			
Stage (0a+0is+I/II+III+IV)	0.461	0.138-1.546	0.210
p53 mutation and PCDH17/Beclin-1 expression	0.112	0.014-0.907	0.033^*^

## Abbreviations

UBC: urinary bladder cancer
MIBC: muscle-invasive bladder cancer
NMIBC: non-muscle-invasive bladder cancer
ISUP: International Society of Urological Pathology
BLCA: bladder cancer
p53: protein 53
p21: protein 21
pRB: RB transcriptional corepressor
PI3K: phosphatidylinositol 3 kinase
TP53: tumor protein p53
PTEN: phosphatase and tensin homologue deleted on chromosome 10
PCDHs: protocadherins
PCDH17: protocadherin 17
FFPE: paraffin-embedded tumors
AJCC: American Joint Committee on Cancer
IHC: immunohistochemistry
PCR: polymerase chain reaction
ATG: autophagy related gene
AMPKα: adenosine monophosphate activated protein kinase α
PTEN/PI3K/AKT/mTOR: phosphatase and tensin homologue deleted on chromosome 10/ phosphatidylinositol 3 kinase/ protein kinase B/ mammalian target of rapamycin
ATG14L-VPS34: autophagy related protein 14L- vacuolar protein sorting
